# Dual Role of Bile Acids on the Biliary Epithelium: Friend or Foe?

**DOI:** 10.3390/ijms20081869

**Published:** 2019-04-16

**Authors:** Leonardo Baiocchi, Tianhao Zhou, Suthat Liangpunsakul, Ilaria Lenci, Francesco Santopaolo, Fanyin Meng, Lindsey Kennedy, Shannon Glaser, Heather Francis, Gianfranco Alpini

**Affiliations:** 1 Liver Unit, Department of Medicine, University of Rome “Tor Vergata”, Viale Oxford 81, 00133 Rome, Italy; baiocchi@uniroma2.it (L.B.); ilaria.lenci@uniroma2.it (I.L.); santopaolofrancesco@gmail.com (F.S.); 2 Department of Medical Physiology, Texas A&M University, College of Medicine 702 SW HK Dodgen Loop, Temple, TX 76504, USA; tzhou@medicine.tamhsc.edu (T.Z.); SGlaser@medicine.tamhsc.edu (S.G.); 3 Richard L. Roudebush VA Medical Center and Indiana University, Gastroenterology, Medicine 1481 W 10th street, Dedication Wing–Room C-7151, Indianapolis, IN 46202, USA; sliangpu@iu.edu (S.L.); mengf@iu.edu (F.M.); linkenn@iu.edu (L.K.); heafranc@iu.edu (H.F.); 4 Division of Gastroenterology and Hepatology, Department of Medicine, Indiana University School of Medicine, 1481 W 10th street, Indianapolis, IN 46202, USA

**Keywords:** bile acids, cholangiocyte, cholestasis, TGR5, ABAT, cholangiocarcinoma

## Abstract

Bile acids are a family of amphipathic compounds predominantly known for their role in solubilizing and absorbing hydrophobic compounds (including liposoluble vitamins) in the intestine. Bile acids also are key signaling molecules and inflammatory agents that activate transcriptional factors and cell signaling pathways that regulate lipid, glucose, and energy metabolism in various human disorders, including chronic liver diseases. However, in the last decade increased awareness has been founded on the physiological and chemical heterogeneity of this category of compounds and their possible beneficial or injurious effects on the biliary tree. In this review, we provide an update on the current understanding of the molecular mechanism involving bile acid and biliary epithelium. The last achievements of the research in this field are summarized, focusing on the molecular aspects and the elements with relevance regarding human liver diseases.

## 1. Bile Acids Biochemistry

Bile acids (BAs) synthesis occurs through cholesterol modification following two principal routes: (a) the classic (neutral) pathway, or (b) the alternative (acidic) pathway [1]. An alternative pathway has been suggested by a study on primary hepatocyte culture [2]. The classical neutral synthesis pathway takes place in the liver and involves the sequential conversion of cholesterol molecules by fourteen enzymes [3]. The first reaction consists of transformation of cholesterol in 7a-hydroxycholesterol by the microsomal cholesterol 7α-hydroxylase (CYP7A1) enzyme. This molecule, a cytochrome P450 oxyreductase enzyme, represents the rate limiting step in BA synthesis. The 7-hydroxycholesterol is then transformed in 7-hydroxy-4-cholesten-3-one by the 3-hydroxy-5-C27-steroid dehydrogenase/isomerase (HSD3B7). For the synthesis of cholic acid (CA) only, a hydroxylation in the 12 position is required in this phase and is accomplished by a sterol 12α-hydroxylase (CYP8B1). The following reaction catalyzed by two cytosolic enzymes, D4–3-oxosteroid-5b-reductase (AKR1D1) and 3α-hydroxysteroid dehydrogenase (AKR1C4), finds the 7α-hydroxy-4-cholesten-3-one and 7α-12 a-hydroxy-4-cholesten-3-one merged together and transform those into 5β-cholestan-3α,7α,12α-triolo and 3α,7α-diolo for the parallel production of CA and chenodeoxycholic acid (CDCA), respectively. The next step involves the mitochondrial sterol 27-hydroxylase (CYP27A1) that oxidizes the side chain with a hydroxyl group at C27, then converts into aldehyde, and finally into a carboxylic acid. The 3α,7α-dihydroxy-5β-cholestanoic acid (DHCA) and 3α,7α,12α-trihydroxy-5β-cholestanoic acid (THCA) are transformed to coenzyme A-esters by two endoplasmic reticulum enzymes: bile acid CoA synthetase (BACS) and very long-chain acyl CoA synthetase (VLCS).

The cholestanoyl-CoAs have their side-chain by peroxisomal enzymes in a beta oxidation process. The last step is represented by bile acid conjugation with either taurine or glycine. This reaction is operated by bile acid CoA-amino acid N-acyltransferase (BAAT). The alternative, or so-called “acidic” pathway, takes this name since acidic metabolites are formed when modification of the cholesterol steroid ring is preceded by side chain oxidation. CYP27A1 is the main enzyme responsible and the limiting step for this reaction, thus transforming cholesterol in 27-hydroxycholesterol and 3β-hydroxy-5-cholestenoic acid. These two are then converted to 7a,27-dihydroxycholesterol and 3b,7a-dihydroxy-5-cholestenoic acid, respectively, by oxysterol 7a-hydroxylase (CYP7B1). These enzymes are mainly present in peripheral tissue. The next steps through this pathway are not completely clear. The liver is the only human organ to own the complete set of enzymes needed to produce BA. The alternative pathway seems to be of minor importance during normal conditions; however, a possible role has been suggested in conditions of liver disease in humans [4].

## 2. BAs Physiology

### 2.1. General Physiologic Functions of BAs

**Figure 1:**
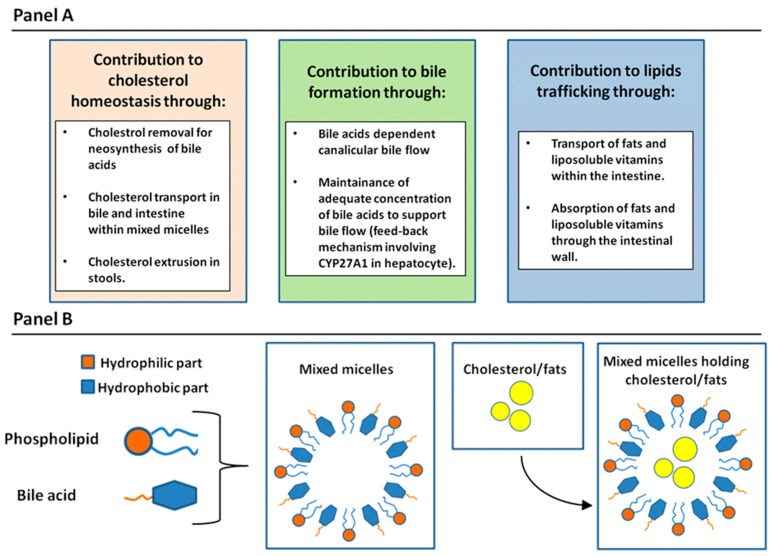
(**A**) The general physiologic function of bile acids is summarized in regard to cholesterol homeostasis, bile secretion, and lipid trafficking to the intestine. CYP27A1 = Sterol 27-hydroxylase. (**B**) Aggregation forms of bile acids with phospholipids and cholesterol and fats in aqueous solution, according to their hydrophilic and hydrophobic portions.

As described by Hoffmann in a 2009 review [5], general physiologic functions of BA may be divided according to target organs. Therefore, there will be effects on: (1) the whole body; (2) the intestines; and (3) the liver. Regarding the systemic effect of bile acids, it is functionally important in cholesterol elimination. BAs are synthesized from cholesterol in the liver and secreted in the bile. The transport of cholesterol by BAs in bile is related to the peculiar physicochemical characteristics of these molecules. In fact, the other two lipidic components in bile, lecithin and cholesterol, have a very low solubility in the aqueous phase. At low concentrations, BAs are in a monomeric form in water; however, when their concentration increases, reaching the so-called “critical micellar concentration”, simple micelles are formed at a concentration ranging from 2 to 20 mM according to the different types of BA. Simple micelles are able to solubilize cholesterol in their hydrophobic core reaching a diameter of 5 angstroms. When phospholipids are incorporated in these structures, mixed micelles are formed in bile, with a diameter up to 30 angstroms and with the capacity to receive three times more cholesterol in comparison with simple micelles [6]. Concerning function in the intestine, their capability to form micellar structures is indeed of paramount importance for the absorption of fats and liposoluble vitamins within the bowel wall. Finally, in the liver, several physiologic functions have been attributed to BAs. Firstly, they play an important role in sustaining canalicular bile flow [7]. A specific BA export pump (BSEP) is present at the level of the hepatocyte canalicular membrane [8]. This molecule belongs to the multi-drug-resistance/transporter associated with antigen processing (MDR/TAP) subfamily of Adenosine Tri-Phosphate binding cassette (ABC) transporters. BSEP is only expressed by the hepatocyte and localized in the cell at the canalicular membrane. The activity of this transporter regulates extrusion of BAs coupled with water in the canalicular space, determining the formation of the “BA-dependent bile flow”. A second important function of BAs is their feedback regulation on their own synthesis in the hepatocytes according to their specific concentration in the cell. This mechanism is obtained by direct regulation of Farnesoid receptor X (FXR) by BAs in hepatocytes. In fact, increased level of BAs in the hepatocytes activates FXR, which is a strong inhibitor of CYP27A1, the rate limiting enzyme in BAs synthesis [9]. As a summary, in Figure 1, general physiologic functions of BAs (Panel A) and aggregation forms of BAs with phospholipids and cholesterol and fats in aqueous solution (Panel B) are reported.

### 2.2. Enterohepatic Circulation of BAs

Enterohepatic circulation of BAs is defined as the cycle of BAs excreted in the bile. Once absorbed by the intestine, these are able to circulate back to the liver via the portal system [10]. The initial driving force in this process is represented by BSEP (as described in the previous paragraph). In the intestine, BAs absorption and translocation in venous blood is regulated by two other transporters: the apical sodium bile acid co-transporter (ASBT), present in the brush border membrane of distal ileum enterocytes, and a heterodimer of two proteins, termed organic solute transporter (OST) alpha and beta [11]. These transporters are responsible for driving BAs through basolateral membranes and into venous blood [12]. The last step consists of BA uptake from the blood by the liver. This is mainly accomplished by the sodium/taurocholate co-transporting polypeptide (NTCP) located in the basolateral membrane of the human hepatocytes [13]. In humans, nearly 2–3 g of BAs are present in the body, while the loss in feces is no more than 10% daily due to the high capacity of recovery by ASBT in the terminal ileum. The amount of BAs left in stools is quickly replaced by liver neo-synthetic pathways. In normal circumstances, the enterohepatic circulation of a molecule of BA is thought to occur 3–4 times a day [14]. 

### 2.3. Physico-Chemical Heterogeneity of BAs with Relevance in Physiopathology

**Figure 2:**
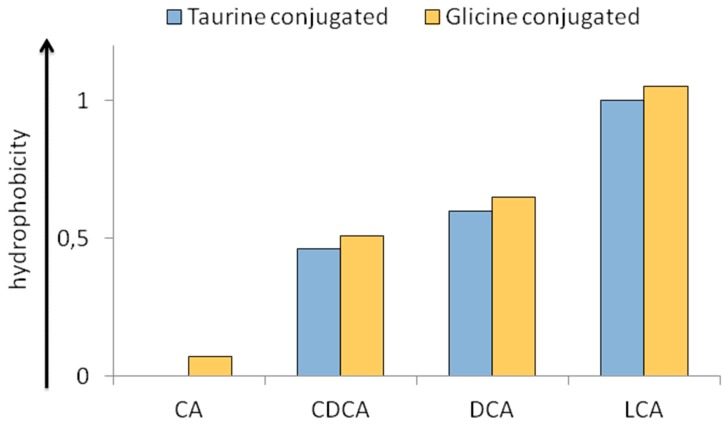
Hydrophobicity index of bile acids (taurine and glycine conjugated) commonly present in human bile, as derived by their partition coefficients in reversed phase high performance liquid chromatography. Hydrophobicity of Tauro CA and Tauro LCA are set to 0 and 1 respectively, by definition. Abbreviations: CA = cholic acid; CDCA = chenodeoxycholic acid; DCA = deoxycholic acid, LCA = lithocholic acid. For complete methods, see reference 20.

The BAs pool circulating in the enterohepatic circulation has to be considered not only in quantitative but also in qualitative terms. The individual BA molecule has a balance between its hydrophobic (steroid) portion and hydrophilic part according to the number and position of different hydroxyl (OH) groups, owning peculiar characteristics in this heterogeneous class of moieties [15]. In this perspective, for instance, human BAs may be ordered according to the hydrophilicity as follows: Cholic acid (CA) > Chenodeoxycholic Acid (CDCA) > Deoxycholic Acid (DCA) > Lithocholic Acid (LCA). Moreover, taurine rather than glycine conjugation confers increased hydrophilicity to the BA molecule. In this sense, the specific hydrophobic-hydrophilic nature of the BA pool, which is a function of its specific qualitative composition, has important effects in the pathological setting [16]. In fact, an increased hydrophobic nature of the BAs pool corresponds to enhanced detergency. Cholestatic liver damage has been historically linked to the retention of detergent BAs in the liver, thus resulting in cellular damage. This concept was demonstrated by a study on isolated hepatocytes and their membranes [17]. From this observation, LCA-induced liver damage has been used by several research teams as a standard model of BA-induced cholestasis [18,19,20]. Finally, specific analytical methods have been designed to assess the specific hydrophobicity index (HI) of a single BA or the BAs pool in bile. In Figure 2, the specific HI of human biliary BAs (either taurine or glycine conjugated) is reported according to their partition coefficients between solid (column) and liquid (mobile) phases in reversed phase high pressure liquid chromatography (HPLC).

In regard to method, an octadecyl silane column is employed with a liquid phase composed of 70% methanol and 30% water. HI of Taurocholic acid (TCA) and Taurolithocholic acid (TLCA) are set to 0 and 1, respectively, by definition [21].

### 2.4. Physiologic Effects of BAs on Biliary Epithelium

The biliary epithelium is composed of bile duct cells or cholangiocytes lining the three-dimensional system formed by the bile ducts [22,23]. Size and function of cholangiocytes are variable according to their location along the biliary tract. Small and large cholangiocytes have been identified and line small and large ducts, respectively. Small cholangiocytes are of cuboidal shape, less specialized, with a high nucleus/cytoplasm ratio. Conversely, large cholangiocytes present a columnar shape, are rich in different cellular organelles, and their cytoplasmic compartment is largely increased in comparison with small cholangiocytes [24,25]. In normal conditions, the most important function of cholangiocytes is the secretin (SCT)-stimulated release of the osmotically active bicarbonate in bile. This mechanism is activated by SCT binding to its receptor (SR) on the basolateral membrane of cholangiocytes, which stimulates the formation of adenosine 3′,5′-cyclic monophosphate (cAMP), and protein kinase A (PKA)-dependent phosphorylation of cystic fibrosis transmembrane conductance regulator (CFTR), resulting in the extrusion of Cl^−^ in the lumen of bile ducts. The Cl^−^ gradient across the plasma membrane activates the apical Cl^−^/HCO_3_^−^ anion exchanger 2 (AE2) and determines excretion of bicarbonate in bile and a concomitant passive influx of water. Through this mechanism, cholangiocytes are thought to determine the so-called bile salt-independent bile flow, accounting for at least 40% of the total flow [26,27]. In normal conditions, SCT and associated intracellular mediators are identified only in large cholangiocytes, so they represent the main actors in bile salt-independent bile flow. On the other hand, small cholangiocytes act as a quiescent population with progenitor phenotypes and are able to replace large cholangiocytes, since the latter are more sensitive to injury. The response of small cholangiocytes to the injury of large cholangiocytes plays a role in the restoration of the normal bile duct cell functional pool after damage [28]. BA signals, on biliary epithelium, act at different levels and are the subject of a review by Jones and co-authors [29]. 

Transmembrane G-protein-coupled receptor (TGR5) is a G-protein-coupled receptor activated by BAs. TGR5 is present in several subcellular locations within the cholangiocyte, including cilia, plasma membranes, intracellular space, vesicles, and endoplasmic reticulum [30]. The localization of TGR5 at the cilia level is of particular interest, since this portion of the cell is exposed to the contents of the bile duct, and thereby is exposed to significant concentration of BAs. In an elegant study, mainly conducted in human cholangiocyte H-69 cells (simian virus 40-transformed human cholangiocytes), the effect of the stimulation of TGR5 was studied in ciliated and non-ciliated cholangiocytes [30]. This study demonstrated that TGR5 is localized in several compartments within cholangiocytes; however, a strong signal was detected mainly in ciliary and nuclear membranes. More interestingly, the activation of this G-protein-coupled receptor resulted in the opposite effect, according to the presence of cilia or not. In fact, while ciliated cholangiocytes decreased their cAMP levels after stimulation of TGR5, the opposite happened in the non-ciliated cells. The decreased cAMP levels were related to binding with a specific G-protein at the base of the cilia, the G alpha-i. Moreover, stimulation of cAMP in non-ciliated cholangiocytes was associated with increased proliferation. Finally, activation of TGR-5 decreased cAMP intracellular levels and proliferation on ciliated cholangiocytes. In contrast, activation of TGR5 stimulated the extracellular regulated protein kinase (ERK) that is a nuclear signaling factor that determines the activation of several genes involved in carcinogenesis [31]. In another study, the important role of TGR-5 in sustaining BA-dependent bile duct cell proliferation and growth was demonstrated [32]. This study was conducted in vitro and in vivo and on TGR-5 knock-out and wild-type mice, and it showed that BA-induced cholangiocyte proliferation was strictly related to TGR-5 expression. Moreover, data coming from isolated cells demonstrated that this effect occurred through the increase of reactive oxygen species (ROS) that in turn activated Rous sarcoma oncogene (cSrc), epidermal growth factor receptor (EGFR), and ERK. An anti-apoptotic effect was also exerted by TGR-5 stimulation in the study. In fact, the use of the CD95 death receptor ligand (CD95l) determined extensive death in TGR-5 knock-out cells and not in control. TGR-5 was also suggested to sustain choleresis, as shown on human gallbladder epithelium [33]. In this study, TGR-5 colocalized with CFTR and stimulated an increase in cAMP and chloride extrusion. This generates secretion of bicarbonate and the so-called bicarbonate-umbrella with protective effects on epithelium. The bicarbonate umbrella has, in fact, been suggested as an important protective mechanism against BAs damage, since the apical secretion of bicarbonate in cholangiocytes determines an alkaline barrier that renders BAs non-protonated and unable to cross membranes [34,35]. Other receptors, expressed at the cholangiocytes level, react with BAs. Sphingosine 1-phosphate receptor 2 (S1PR2) is stimulated by BAs and induces bile duct cell proliferation through the ERK/protein Kinase B (AKT) pathway [36]. FXR, even if preferentially expressed in hepatocytes, has also been detected in bile duct cells. Simulation of FXR in cholangiocytes has been related to increased expression of fibroblast growth factor (FGF) 15/19 and inhibition of sterol Cyp27A1 that is the rate-limiting enzyme for the acidic pathway of BA biosynthesis. On the basis of these results, a possible cholangiocyte regulation of BA synthesis, through the acidic pathway, has been hypothesized [37].

Finally, a specific transporter for BAs, the Na-dependent apical bile acid transporter (ABAT), was identified at the end of the millennium, on the apical portion of the cholangiocytes [38,39]. The possibility that unprotonated, unconjugated BAs should be reabsorbed by the biliary tract was already hypothesized to justify the excessive choleresis stimulated by a certain type of BAs, and has been termed the cholehepatic shunt [40]. After the identification of ABAT, it was shown that internalization of BAs in the biliary epithelium is a key factor for several processes, including proliferation, differentiation, secretion, and apoptosis [41].

### 2.5. Protective Effects of BAs on Biliary Epithelium

Adequate presence of BAs in bile is of paramount importance to prevent pathological changes in the biliary tract. In one study, 24 h of BA depletion in rats was shown to depress SR-stimulated bile secretion, SR-induced increase in intracellular cAMP, and the normal proliferative activity of cholangiocytes, as demonstrated by the reduction of protein cellular nuclear antigen (PCNA) in cells [42]. Moreover, ABAT, which is present in cholangiocytes that regulate the internalization of BAs, was depressed. Interestingly, when BA-depleted rats were infused with TCA (sodium salt) at 1 micro mol/min/kg body weight, normal condition of the biliary tree was restored. This study helped clarify the behavior of ABAT in normal and pathological conditions, such as BAs depletion.

Physiological BAs were also found to be protective in other pathologic settings, such as vagotomy, CCl_4_-induced liver fibrosis, and hepatic artery ligation in rats. Vagotomy has been demonstrated to induce cholangiocyte apoptosis and loss of proliferation. These effects were related to reduced intracellular cholangiocyte cAMP levels, but preservation of cAMP levels by administration of forskolin prevents the effects of vagotomy on the biliary tract [43].

In this context, when vagotomy was performed in bile duct ligated (BDL) rats (the latter is a strategy to enhance proliferative activities of bile duct cells), TCA feeding was able to mitigate cholangiocyte apoptosis and the vagotomy-induced inhibition of cholangiocyte proliferation and secretin-stimulated ductal secretion [43]. Prevention of apoptosis by TCA was phosphatidylinositol 3-kinases (PI3K)/AKT, mediated as observed in other cell lines [44]. The beneficial effects of TCA were also demonstrated in a model of carbon tetrachloride (CCl_4_)-mediated bile duct damage. CCl_4_ triggers significant damage to cholangiocytes after a single dose by gavage [45]. Interestingly, the injurious effects are restricted mainly in the physiologically-active large cholangiocytes. The explanation for this phenomenon was explained by the fact that CCl_4_ injury is related to its metabolic reactive intermediates, catalyzed by cytochrome P450 [46,47], but small cholangiocytes lack this particular enzyme. TCA abolished the damaging effects of CCl_4_ [48]. The mechanisms regulating this effect were similar to those reported when injury was induced by vagotomy. In fact, a clear reduction of apoptosis and preserved proliferation and ductal secretion activities were observed. At the molecular level, the beneficial effects were associated with increased intracellular levels of cAMP and activation of PI3K-AKT-dependent survival pathway. The inhibitor of PI3 Kinase pathway, wortmannin, abolished the positive effect of TCA feeding. 

**Figure 3:**
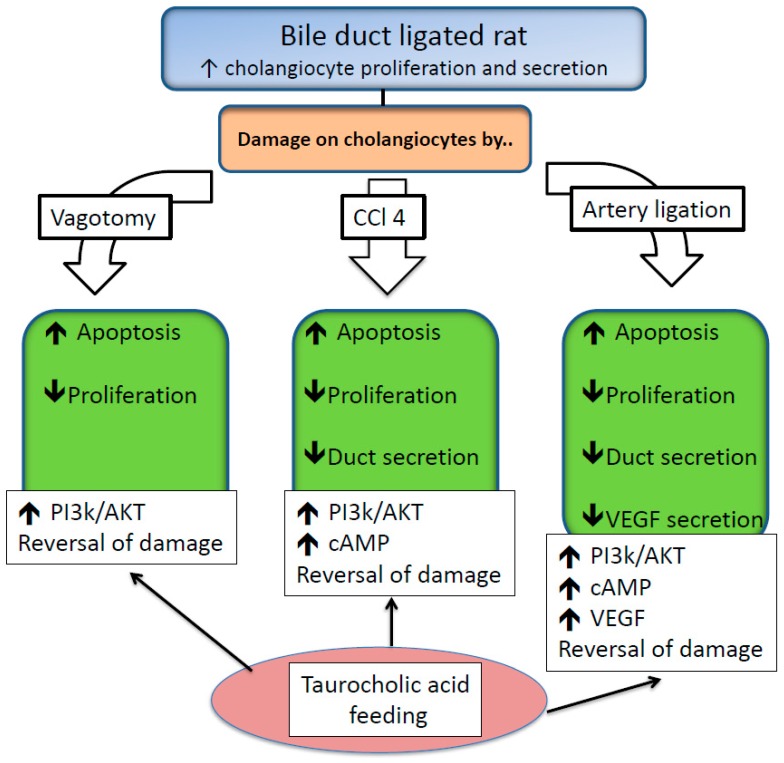
Acid feeding reversal of damage and the corresponding mechanisms in different models of cholangiocyte injury in bile duct ligated rats. Abbreviations: CCL4 = carbon tetrachloride; PI3k/AKT = phosphatidylinositol 3-kinase/protein kinase B; cAMP = cyclic adenosine monophosphate; VEGF = vascular endothelial growth factor. Symbols: ↑ = increase; ↓ = decrease.

Hepatic artery ligation represents another model of biliary damage resembling human disease associated with vascular complications after liver transplantation or other surgical procedures [49,50]. Normal bile duct cells are nourished by the hepatic artery through the peri-biliary plexus (PBP). Proliferation of the biliary tract is associated with a parallel increase and extension of the PBP, thus suggesting cross-talk between these two anatomical structures. In fact, large cholangiocytes express the protein for vascular endothelial growth factor (VEGF)-A, secrete VEGF, and express the VEGF receptor subtypes VEGFR-2 and VEGFR-3 but not VEGFR-1 [51]. Moreover, VEGF secretion is increased during BDL-induced cholestasis in rats and stimulates cholangiocyte proliferation via an autocrine mechanism. In the BDL rat model, ligation of the hepatic artery: (i) caused disappearance of the PBP, (ii) increased apoptosis and impaired cholangiocyte proliferation and SCT-stimulated ductal secretion, and (iii) decreased cholangiocyte VEGF secretion [52]. TCA feeding in these animals is able to counteract all these effects. In regard to cholestasis induced by artery ligation, the TCA beneficial effect involved the PI3-K/AKT-dependent signaling mechanism, since it was blocked by wortmannin. In addition, TCA feeding was able to restore appropriate VEGF secretion by cholangiocytes, thus maintaining a functional VEGF autocrine loop, suggesting this as a possible beneficial effect in favor of TCA [53]. In Figure 3, the mechanisms of TCA protective effects in vagotomy, CCl_4_-induced liver fibrosis, and hepatic artery ligation in BDL rats are reported.

In this previous part of the paragraph, the protective effect of physiological BAs was reported (TCA is the most represented BA in rodent bile, corresponding in humans to the glyco-conjugated form Glycocholic acid). However, a natural BA, the 3α,7β-dihydroxy-5β-cholan-24-oic, Ursodeoxycholic acid (UDCA), not present in significant proportions in rodent or human bile in the normal state, captured the scientific attention for its protective properties in cholestatic conditions. UDCA now represents the first line of therapy for the most frequent cholestatic chronic human liver disease, primary biliary cholangitis (PBC) [54]. 

In patients with this affliction, UDCA (10–15 mg/Kg/day) has been demonstrated to improve liver function tests, to delay fibrosis, and to increase survival [55,56]. UDCA use in Primary Sclerosing Cholangitis (PSC), another relevant chronic colestatic disease in human, remains more controversial. While this therapy is effective in reducing liver enzymes [57], the lack of effects on long-term survival and the evidence of increased risk of a worse outcome in subjects assuming UDCA at high doses (28–30 mg/kg/day) does not suggest its routine use in PSC [58]. However, that is to underline that this disease, even if included in the chapter of human chronic cholestasis, remains infrequent and poorly understood. In fact, it is characterized by a strong inflammatory component (usually present for several years before the onset of cholestasis) causing macroscopic strictures in the biliary tract, not amenable whatsoever to medical treatment, which commonly requires endoscopic or surgical revision. 

The beneficial effects of UDCA during cholestasis have been related to different mechanisms, including: (i) stimulation of altered hepatocyte secretion; (ii) consolidation of the bicarbonate-umbrella at the apical domain of cholangiocytes; (iii) modification of hydrophobic–hydrophilic balance of the BAs pool, determining lower overall detergence of bile; and (iv) anti-apoptotic effects [59]. In regard to bile duct cells, a study was conducted where BDL rats were fed with either UDCA or its taurine conjugate, TUDCA [60]. The main results of this study were that UDCA or TUDCA feeding: (i) down-regulated the increased proliferative activities; and (ii) inhibited the increased ductal secretion induced by cholestasis. These effects were not related to increased apoptosis or necrosis in cholangiocytes. Moreover, down-regulation of ABAT was demonstrated during UDCA or TUDCA feeding. The cell proliferation related to hormones or growth factors frequently requires protein kinase C (PKC) activation [61], and BAs modulation of PKC was demonstrated in hepatocytes [62,63]; this study also evaluated intra-cellular Ca2^+^ concentration and PKC alpha activity. Results demonstrated that PKC alpha activation and increased intra-cellular Ca2^+^ concentration were required for UDCA and TUDCA effects on cholangiocyte proliferation and secretion.

Another study examined the effects of UDCA or TUDCA feeding in the same model, as described previously, of BDL rats undergoing vagotomy [64]. This research aimed mainly to assess the effects of UDCA in a model of cholestasis characterized by loss of bile duct cells, and specifically assessed Ca^2+^ signaling, and PI3K and mitogen-activated protein kinase (MAPK) pathways that have been previously demonstrated to be related to UDCA beneficial effects in hepatocytes [65,66]. Results demonstrated that UDCA or TUDCA feeding was able to reverse bile duct loss, and these effects were related to increased intra-cellular Ca2^+^ concentration and activation of the PI3K and MAPK pathway. 

Recently, a new piece of novel evidence was added on the UDCA beneficial effect on cholangiocytes during cholestasis. A study conducted in Multi-Drug Resistance (Mdr)2^−/−^ mice (a model mimicking the pathological features of human primary sclerosing cholangitis (PSC) and on liver tissue sections of patients with PSC analyzed the effect of UDCA on inflammatory response [67]. A paramount importance was previously described in regard to the role of mast cells in promoting damage surrounding bile ducts and in the increase in histamine levels, proliferation, and hepatic fibrosis in the Mdr2^−/−^ mice model [68]. With this background, the analysis of inflammation after UDCA was greatly improved. In fact, mast cell infiltration was reduced, as well as biliary damage, reactive proliferation, and fibrosis. Local release of histamine was also reduced by UDCA. Interestingly, inflammation and mast cell infiltration were reduced in liver sections of PSC patients treated with UDCA in comparison with the subjects not receiving this therapy. This study represents the first evidence of a possible role of UDCA in modulating inflammation through regulation of mast cell activity.

### 2.6. Injurious Effects of BAs on Biliary Epithelium

In the past, it was thought that BAs could cause damage in the liver during cholestasis due to their detergent cytolytic properties or by apoptosis [69,70]. Further studies, however, expanded this vision, suggesting that the possible BAs injurious effects are more complex, not linked to intrinsic toxicity but related to other mechanisms, including the possible activation of inflammatory pathways [71]. With regard to cholangiocytes, a first study, undertaken two decades ago, evaluated conjugated and unconjugated BAs toxicity in isolated bile ducts and isolated-perfused rat liver [72]. The authors demonstrated that unconjugated, more hydrophobic BAs were able to determine ultra-structural alterations, including swelling with rarefaction of the matrix in the mitochondria and dilatation of the cisternae of the Golgi apparatus. Interestingly, the integrity of cell membranes remained unaltered, thus suggesting a mechanism of damage not related to a direct detergent effect of BAs. Moreover, no signs of cytotoxicity were observed in the perfused rat liver system, suggesting that removal and transport of BAs in intact liver prevent possible BAs toxicity “in vivo”. 

A following study focused on the possible biliary damage induced by LCA (the most hydrophobic BA) by feeding in mice [73]. On a morphological base a destructive cholangitis characterized by peri-ductular fibrosis, partial obstruction and bile infarcts were observed. In order to explain the mechanism of bile duct injury in this setting, the authors hypothesized a direct LCA cytotoxicity for its precipitation in the cause of the intrinsic scarce solubility. The possible contribution of hepatocytes in cholestasis was considered irrelevant, since the expression of hepatocyte canalicular transporters was similar to what was observed in the BDL rat model [74]. Since LCA feeding-induced destructive cholangitis was characterized by neutrophils infiltration, a further study examined the possible role of these cells in this model of biliary damage [75]. In fact, in the cholestatic liver injury model of BDL rodents, resembling pathological aspects of LCA-feeding damage, beta-2 integrin (CD-18) deficient mice that are lacking an efficient extracellular matrix binding property for neutrophils, exhibited lower cholestatic damage in comparison with controls [76]. However, when a similar approach was used in the LCA cholestatic mice, employing intercellular adhesion molecule-1 (ICAM-1) deficient mice (that do not express this protein fundamental for neutrophil toxicity), cholestasis was not affected by reduction in neutrophil activity. In conclusion, these data suggested that more hydrophobic BAs are able to directly induce cholestatic liver damage regardless of neutrophil tissue infiltration. At present, the clear mechanism of LCA feeding-induced destructive cholangitis remains an open issue and a target for possible future research. Possible contribution to bile duct injury is, however, not to be considered a requisite of more hydrophobic BAs only. In fact, TCA (the most represented, physiologic BA in rat bile) has also been reported to be protective in several injury models, exerting important regulatory properties for bile duct proliferation and secretion that might be detrimental in some cholestatic conditions. In this perspective, TCA signaling has been advocated as the most important trigger for BDL-induced damage in rodents [77]. In a study, in fact, it was demonstrated that S1PR2 is the predominant S1P receptor expressed in cholangiocytes. Several days of BDL in mice determined an increased expression of S1PR2 almost exclusively in cholangiocytes within the liver. Since S1PR2 activation is a key factor for progression of cholestatic damage in BDL mice (demonstrated by a reduce injury in S1PR2 knockout mice in the same study) and TCA is able to greatly stimulate this receptor, it was concluded that this BA may contribute (in physiologic condition and concentration) to BDL mice liver damage. Intracellular mediators of inflammatory damage, induced by S1PR2 stimulation, are then represented by the ERK1/2/AKT/nuclear factor kappa-light-chain-enhancer of B cell (NF-kB) axis, with increased Cyclooxygenase 2 activity at the biliary level.

Another chapter of pathology in which BAs interaction with cholangiocytes may play a major role is represented by human cholangiocarcinoma (CCA). CCA originates from bile duct cells and is the second most frequent primary liver tumor in humans [78]. The rise of CCA in certain chronic cholestatic conditions, such as primary sclerosing cholangitis (PSC), has captured the attention on BAs as a possible oncogenic factor. In fact, in experimental models of bile duct obstruction in rodents, an increased progression of CCA has been recorded [79]. Another experimental study, examining more in-depth BAs and CCA cell interaction, evidenced a different effect between conjugated and unconjugated BAs on CCA growth [80]. In fact, conjugated BAs only stimulated CCA growth, activating the NF-kB pathway. Dysregulation of this pathway has been linked to several pathological sequelae of the cell, including tumorigenesis. A downstream effector of NF-kB is, in fact, interleukin-6 (IL6), which has been demonstrated to be increased in several epithelial tumors. With this background, conjugated BAs stimulation of NF-kB is now considered an important mechanism of CCA growth, and experimental approaches aimed at repressing this pathway have demonstrated reduced tumor growth significantly [81,82,83]. In addition, stimulation of S1PR2 may also play a role in this setting. In a study of CCA cell lines, it was demonstrated that activation of S1PR2 (a receptor strongly stimulated by conjugated BAs, such as TCA) promotes proliferation and neoplastic cell migration and spreading. These effects were obtained by a S1PR2-dependent activation of ERK1/2- and AKT-signaling pathways [84]. 

Another mechanism by which BAs seems to stimulate CCA growth is direct interaction with the TGR-5 receptor on the apical membrane of cholangiocytes, as described in paragraph 4. TGR-5 expression has been demonstrated to be increased in CCA in comparison with surrounding tissue in human [36]. Moreover, stimulation of this receptor is associated in vitro with increased proliferation, migration, and mitochondrial energy expenditure of neoplastic CCA cells. These results were not replicated in an in vivo orthotopic mouse model of CCA, in which the specific TGR5 agonist INT-777 was employed.

**Figure 4:**
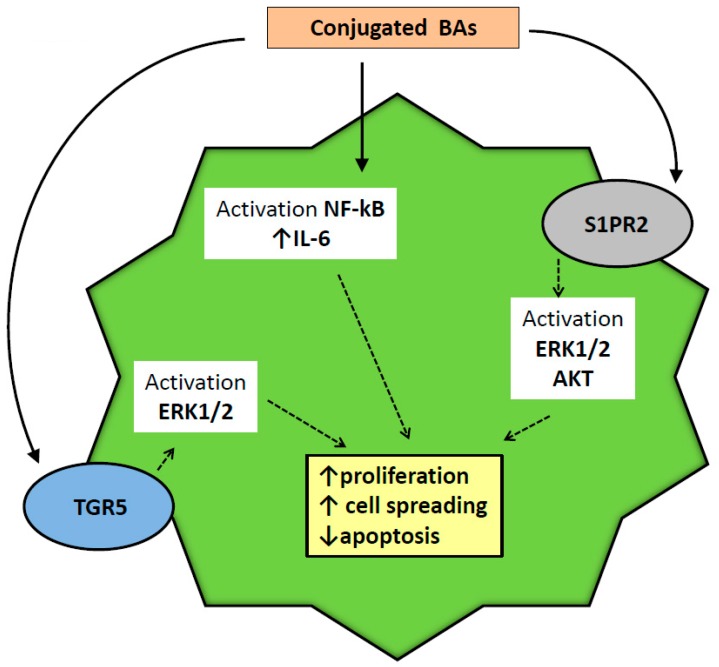
Bile-acid-activated growth and spreading of cholangiocarcinoma. Abbreviations: BAs = bile acids; NF-kB = nuclear factor kappa-light-chain-enhancer of B cells; IL- 6= interleukin- 6; S1PR2 = sphingosine 1-phosphate receptor 2; ERK = extracellular regulated protein kinase; AKT = protein kinase B; TGR5 = transmembrane G protein coupled receptor. Symbols: ↑ = increase; ↓ = decrease. Solid arrows: initial step activated by bile acids; Dotted arrows: following molecular steps after stimulation by bile acids.

In conclusion, BAs may have an important role in the genesis and growth of CCA. Their role, however, should not be linked to a direct oncogenic effect but to their potential as a growth factor interacting with specific pathways, such as NF-kB and TGR-5. A schematic summary of the pathways involved in BA stimulation of cholangiocarcinoma proliferation and cell spreading is depicted in Figure 4.

## 3. Conclusions

Recent advances in the study of BAs and biliary epithelium have revealed that these molecules not only play a fundamental role in bile formation and secretion, but are also important in protective and injurious processes involving the biliary tract. The identification of a specific apical transporter (ABAT) and receptor (TGR5) for BAs in cholangiocytes has represented a major turning point in the understanding of the interaction between these cells and bile. Our knowledge in this field has been implemented by the findings that BAs are also able to modulate proliferation, apoptosis resistance, and preservation of ductal bile secretion during injury. On the other hand, in particular conditions, such as during neoplastic disease of the biliary tract, antiapoptotic and proliferative effects of BAs on biliary epithelium may result in detrimental effects, while more hydrophobic BAs, such as LCA, have been demonstrated to be deleterious for biliary epithelium. The pathways involved in injury and protection by BAs on the biliary tract remain to be completely clarified at present. Modulation of these axes may warrant, in the future, an increase comprehension in regard to genetic, acute, and chronic cholestatic human liver diseases, and possibly suggests therapeutic strategies for these afflictions. Finally, manipulation of steroid moieties and production of synthetic BAs has been pursued for therapeutic purposes with good results. In fact, Obeticholic acid (6α-ethyl-chenodeoxycholic acid), a semi-synthetic BA identified as a strong activator of FXR; starting from 2016, it was approved for the treatment of human PBC, both in the United States and Europe. Obeticholic acid therapy (5–10 mg day) is indeed beneficial, reducing cholestasis indices in PBC patients, including when they are in a concomitant treatment with UDCA [85,86]. Identification and synthesis of artificial BAs with enhanced activity for cholangiocytes receptors, such as TGR5 or S1PR2, may be useful to increase our knowledge on bile duct cells activities and a possible opportunity for human therapy. 

## Abbreviations

ABC	Adenosine Tri-Phosphate binding cassette
AE2	apical Cl^−^/HCO_3_^−^ anion exchanger 2
ASBT	apical sodium bile acid co-transporter
AKR1C4	3a-hydroxysteroid dehydrogenase
AKR1D1	D4–3-oxosteroid-5b-reductase
BAAT	bile acid CoA-amino acid N-acyltransferase
BACS	bile acid CoA synthetase
BDL	bile duct ligated, (BSEP
cAMP	adenosine 3′,5′-cyclic monophosphate
CCA	cholangiocarcinoma
CCl_4_	carbon tetrachloride
CDCA	chenodeoxycholic acid
CYP27A1	Sterol 27-hydroxylase
CFTR	cystic fibrosis transmembrane conductance regulator
CYP7B1	oxysterol 7a-hydroxylase
CYP8B1	12a-hydroxylase
cSrc	Rous sarcoma oncogene
DCA	Deoxycholic Acid
DHCA	3a,7a-dihydroxy-5b-cholestanoic acid
EGFR	epidermal growth factor receptor
ERK	extracellular regulated protein kinase
FXR	Farnesoid receptor X
HI	hydrophobicity index
HPLC	high pressure liquid chromatography
ICAM-1	intercellular adhesion molecule-1
IL6	interleukin-6
LCA	Lithocholic Acid
MAPK	mitogen-activated protein kinase
Mdr	Multi-Drug Resistance
MDR/TAP	multi-drug-resistance/transporter associated with antigen processing
NTCP	sodium/taurocholate co-transporting polypeptide
OST	organic solute transporter
PBP	peri-biliary plexus
PBC	primary biliary cholangitis
PCNA	protein cellular nuclear antigen
PI3K	phosphatidylinositol 3-kinases
PKA	protein kinase A
PKC	protein kinase C
PSC	primary sclerosing cholangitis
ROS	reactive oxygen species
SCT	secretin
S1PR2	Sphingosine 1-phosphate receptor 2
SR	secretin receptor
TCA	Taurocholic acid
TGR5	Transmembrane G protein coupled receptor
THCA	3 a,7a,12a-trihydroxy-5b-cholestanoic acid
TLCA	Taurolithocholic acid
TUDCA	Tauroursodeoxycholic acid
UDCA	Ursodeoxycholic acid
VEGF	vascular endothelial growth factor
VLCS	very long-chain acyl CoA synthetase
